# Clinical management and outcomes of patients with Hermansky-Pudlak syndrome pulmonary fibrosis evaluated for lung transplantation

**DOI:** 10.1371/journal.pone.0194193

**Published:** 2018-03-16

**Authors:** Souheil El-Chemaly, Kevin J. O’Brien, Steven D. Nathan, Gerald L. Weinhouse, Hilary J. Goldberg, Jean M. Connors, Ye Cui, Todd L. Astor, Philip C. Camp, Ivan O. Rosas, Merte Lemma, Vladislav Speransky, Melissa A. Merideth, William A. Gahl, Bernadette R. Gochuico

**Affiliations:** 1 Division of Pulmonary and Critical Care Medicine, Brigham and Women’s Hospital, Boston, Massachusetts, United States of America; 2 Office of the Clinical Director, National Human Genome Research Institute, National Institutes of Health, Bethesda, Maryland, United States of America; 3 Advanced Lung Disease and Transplant Program, Inova Fairfax Hospital, Falls Church, Virginia, United States of America; 4 Division of Hematology, Brigham and Women’s Hospital, Boston, Massachusetts, United States of America; 5 Division of Pulmonary & Critical Care Medicine, Massachusetts General Hospital, Boston, Massachusetts, United States of America; 6 Division of Thoracic Surgery, Brigham and Women’s Hospital, Boston, Massachusetts, United States of America; 7 National Institute of Biomedical Imaging and Bioengineering, National Institutes of Health, Bethesda, Maryland, United States of America; 8 Medical Genetics Branch, National Human Genome Research Institute, National Institutes of Health, Bethesda, Maryland, United States of America; University of Louisville, UNITED STATES

## Abstract

Pulmonary fibrosis is a progressive, fatal manifestation of Hermansky-Pudlak syndrome (HPS). Some patients with advanced HPS pulmonary fibrosis undergo lung transplantation despite their disease-associated bleeding tendency; others die while awaiting donor organs. The objective of this study is to determine the clinical management and outcomes of a cohort with advanced HPS pulmonary fibrosis who were evaluated for lung transplantation. Six patients with HPS-1 pulmonary fibrosis were evaluated at the National Institutes of Health Clinical Center and one of two regional lung transplant centers. Their median age was 41.5 years pre-transplant. Three of six patients died without receiving a lung transplant. One of these was referred with end-stage pulmonary fibrosis and died before a donor organ became available, and donor organs were not identified for two other patients sensitized from prior blood product transfusions. Three of six patients received bilateral lung transplants; they did not have a history of excessive bleeding. One patient received peri-operative desmopressin, one was transfused with intra-operative platelets, and one received extracorporeal membrane oxygenation and intra-operative prothrombin complex concentrate, platelet transfusion, and desmopressin. One transplant recipient experienced acute rejection that responded to pulsed steroids. No evidence of chronic lung allograft dysfunction or recurrence of HPS pulmonary fibrosis was detected up to 6 years post-transplant in these three lung transplant recipients. In conclusion, lung transplantation and extracorporeal membrane oxygenation are viable options for patients with HPS pulmonary fibrosis. Alloimmunization in HPS patients is an important and potentially preventable barrier to lung transplantation; interventions to limit alloimmunization should be implemented in HPS patients at risk of pulmonary fibrosis to optimize their candidacy for future lung transplants.

## Introduction

Pulmonary fibrosis is a progressive interstitial lung disease that develops in people with Hermansky-Pudlak syndrome (HPS), a rare autosomal recessive disease characterized by defective biogenesis of lysosome-related organelles [1]. Other clinical manifestations of HPS include oculocutaneous albinism, bleeding due to a platelet storage pool deficiency, and granulomatous colitis [2, 3]. Ten subtypes of HPS have been reported; three HPS subtypes are associated with fibrotic lung disease [4–12]. Generally, middle-aged adults with HPS-1 or HPS-4 and children or adults with HPS-2 are at risk of developing pulmonary fibrosis [2, 13–15]. No medical therapies approved by the Food and Drug Administration are available for the management of HPS pulmonary fibrosis, and respiratory failure due to fibrotic lung disease is a major cause of death for many patients with HPS-1 [16, 17].

Lung transplantation has been successfully performed in patients with severe HPS pulmonary fibrosis, and remains the only available therapy for such individuals [18–20]. A potential contraindication to performing surgery in patients with HPS is their tendency to bleed due to a deficiency of platelet dense bodies. Fortunately, treatment with intravenous desmopressin or transfusion with normal platelets provides temporary correction of their qualitative platelet dysfunction. Thus, the bleeding diathesis associated with HPS is not a major impediment to performing surgery, including lung transplantation.

Although lung transplantation is a therapeutic option for HPS pulmonary fibrosis, limited information is available regarding lung transplants for patients with HPS. We report the pre-transplant evaluations, clinical management and outcomes of 6 patients with HPS pulmonary fibrosis who were referred to 2 independent regional lung transplantation centers in the United States.

## Materials and methods

### Subject selection

Patient subjects provided written informed consent and were enrolled in protocol 95-HG-0193 and/or 04-HG-0211, which were approved by the Institutional Review Board of the National Human Genome Research Institute. Patients were evaluated at the National Institutes of Health (NIH) Clinical Center and Brigham and Women’s Hospital or Inova Fairfax Hospital for suitability for lung transplant. Retrospective chart review was conducted and data with identifiers were accessed by an investigator with Institutional Review Board approval at Brigham and Women’s Hospital (protocol number 2014P000189), who waived the need for consent. This study did not involve the use of donated tissue or organs. The diagnosis of HPS was confirmed by platelet whole mount electron microscopy, which demonstrated absent platelet delta granules, and by molecular testing, which identified genetic mutations in *HPS1* [2]. The diagnosis of pulmonary fibrosis was established in patients with HPS by identifying characteristic findings on high-resolution computed tomography scans of the chest [13].

### Pulmonary function testing and imaging

Pulmonary function tests as well as conventional and high resolution computed tomography scans of the chest were performed at the NIH Clinical Center as previously described [21].

### Screening for circulating antibodies

Candidates for lung transplant at Brigham and Women’s Hospital were evaluated for the presence of class I and class II anti-HLA antibodies using standard protocols [22]. Calculated Panel of Reactive Antibodies (cPRA) was measured using the Organ Procurement and Transplantation Network (OPTN) website cPRA calculator for class I anti-HLA antibodies.

### Query of UNOS database

The United Network for Organ Sharing (UNOS) administers the OPTN to collect and manage data regarding transplant events in the United States. The UNOS database was queried for patients with HPS pulmonary fibrosis listed, transplanted or removed from the waitlist between January 1, 2005 and August 31, 2014.

## Results

### Patient characteristics

**Fig 1:**
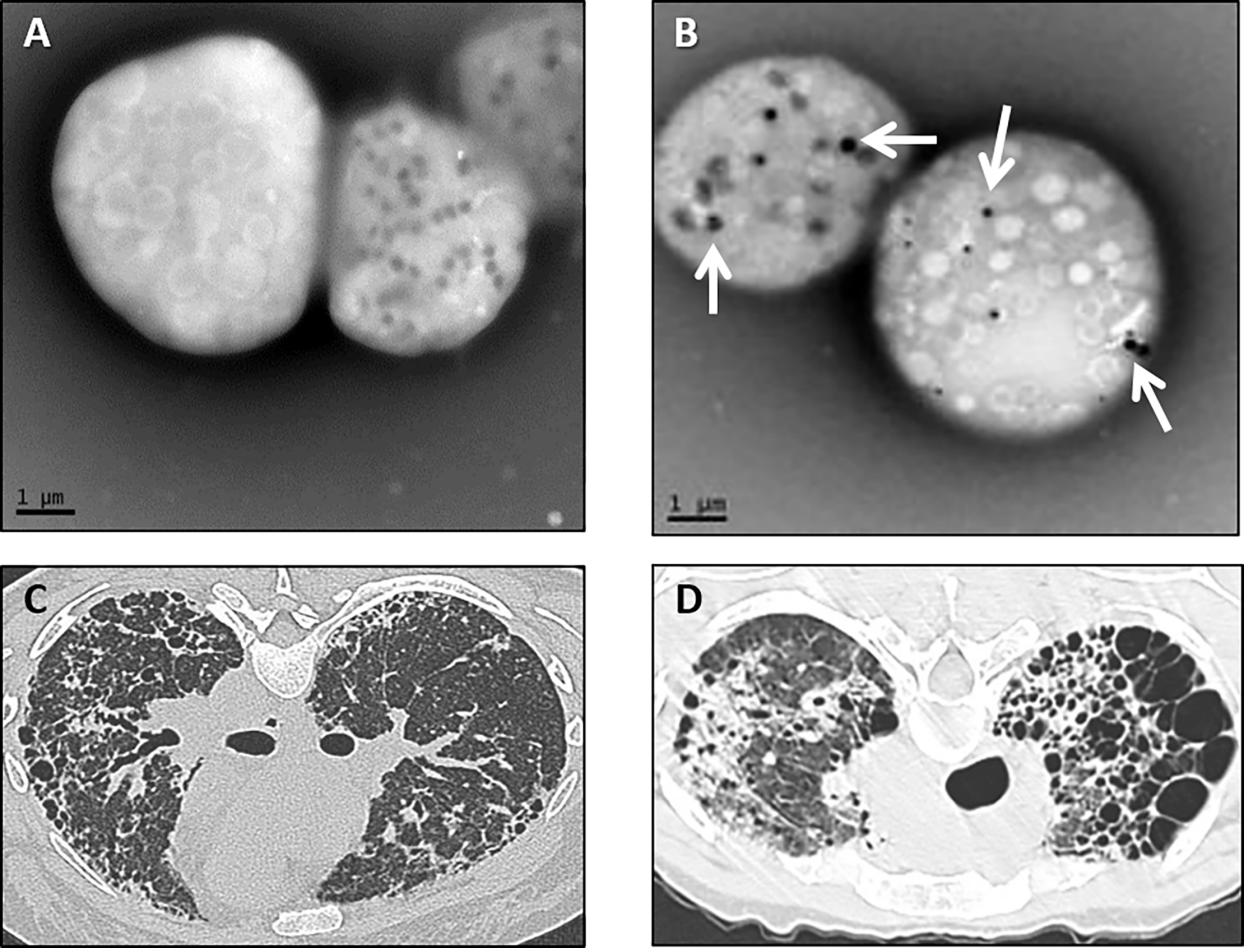
Electron micrograph of platelets and chest computed tomography scans in patients with Hermansky-Pudlak syndrome. Representative whole-mount electron micrographs of platelets from a patient with Hermansky-Pudlak syndrome (A) and normal platelets (B) are shown (bar = 1 micrometer). Normal platelets contain delta granules (arrows), and platelets from patients with HPS are devoid of delta granules. Representative computed tomography scan of the chest from one patient with Hermansky-Pudlak syndrome pulmonary fibrosis showing diffuse bilateral parenchymal fibrosis with honeycombing and loss of lung volume (C) and from another patient with cystic lung destruction and upper lobe predominance of disease (D).

Six patients with HPS pulmonary fibrosis were evaluated for lung transplantation between 2007 and 2015 (Table 1 and S1 Table). The median age at the time of evaluation was 41.5 years (range 28–50 years); 2 of 6 patients were male. None of the patients were smokers. Whole mount electron microscopy demonstrated absent platelet delta granules in all patients (Fig 1A and Fig 1B). Molecular testing of genomic DNA showed that 5 patients had homozygous mutations with a 16-base pair duplication in exon 15 of *HPS1*, which is commonly found in individuals with HPS-1 of Puerto Rican heritage [2]. One patient had a compound heterozygous mutation in *HPS1* that included an allele with this same 16-base pair duplication.

**Table 1:** Patient characteristics.

	Patient 1	Patient 2	Patient 3	Patient 4	Patient 5	Patient 6
Age (years)	50	44	38	50	39	28
Skin cancer	SquaCCa	No	SquaCCa	No	No	No
Colitis	No	No	No	No	No	No
Initial FVC	59	46	58	35	46	14
Initial 6MWT	562	375	427	254	558	134
Oxygen	No	No	No	Yes	No	Yes
PAP	37	30	50	27	74	47
LAS	65	95	76	41	90	91
Outcome	Transplant	Death on waitlist	Death on waitlist	Transplant	Transplant	Death on waitlist

FVC, forced vital capacity (% predicted)

PAP, pulmonary artery systolic pressure (mm Hg)

LAS, lung allocation score

SquaCCa, squamous cell carcinoma

### Pre-transplant evaluations

Pulmonary function testing showed that the median forced vital capacity (FVC) was 48.5% of predicted (range 14–59% of predicted). Six-minute walk testing found that 4 of 6 patients ambulated less than 500 meters (mean 385 meters; range 134–562 meters). At their initial evaluations, 2 of 6 patients received supplemental oxygen, and their FVC values were 35% predicted and 14% predicted. All patients developed progressive HPS pulmonary fibrosis and required oxygen supplementation prior to lung transplantation or death. High-resolution computed tomography scans demonstrated characteristic features of HPS pulmonary fibrosis, including loss of lung volume, reticulation, honeycombing, traction bronchiectasis, and ground glass opacities (Fig 1C) [13]. While one patient had an upper lobe predominance of disease with fibrosis and lung cysts (Fig 1D), the other 5 patients had diffuse parenchymal lung fibrosis.

Two patients had histories of squamous cell carcinoma of the skin. The malignancy of one patient was excised 4 months prior to listing for lung transplant. A second patient had multiple squamous cell skin carcinomas, which were excised 3, 4 and 5 years before listing for lung transplant. No patient had a history of non-dermatologic malignancy or active colitis.

On hematologic evaluation, two patients had histories of bleeding episodes and required packed red blood cell and/or platelet transfusions; 4 patients had not received blood products (Table 2). All patients had normal platelet counts. Two patients had received desmopressin, and one was previously treated with aminocaproic acid. One of four patients tested had a low level of von Willebrand factor. Screening for circulating anti-HLA antibodies using cPRA testing was performed in 5 of 6 patients. Three patients who never received blood products had cPRA scores of zero. In the 2 patients who had a previous history of red blood cell and/or platelet transfusions, circulating anti-donor specific antibodies were detected at high levels, indicating prior alloimmunization. One patient received platelet transfusions 2, 9, and 10 years before cPRA measurement, and the other patient was transfused with platelets and red blood cells within a year of cPRA measurement. In the former patient, the broad array of antibodies present was directed at 100% of the potential donor pool.

**Table 2:** Summary of hematology history and antibody panel reactivity.

	Patient 1	Patient 2	Patient 3	Patient 4	Patient 5	Patient 6
History of bleeding	no	yes	yes	no	no	no
Platelet count (K/microliter)	334	279	464	393	309	168
History of desmopressin	no	no	yes	yes	no^a^	no
vWF	normal	normal	low	n/a	normal	n/a
History of transfusions	no	yes^b^	yes^c^	no	no	no
Class I antibody reactivity	negative	positive	positive	negative	negative	n/a
Class II antibody reactivity	negative	positive	positive	negative	negative	n/a
cPRA	0	41	100	0	0	n/a

cPRA, calculated Panel of Reactive Antibodies

n/a, not available or not performed

vWF, von Willebrand factor

^a^ treatment with aminocaproic acid

^b^ 20 units of platelets in total

^c^ 8 units of platelets and 2 units of packed red blood cells

### Clinical outcomes of patients

Three of 6 patients died prior to receiving a lung transplant, and the primary cause of death was respiratory failure. One patient referred with very severe lung disease had an FVC of 14% of predicted, pulmonary artery systolic pressure (PAP) of 47 mm Hg, and lung allocation score (LAS) of 91. This patient died after being active on the lung transplant wait list for 3 months. The other 2 patients referred to a lung transplant center with FVCs of 46% and 58% of predicted died without receiving transplants. Values of PAP were 30 and 50 mm Hg, and of LAS were 95 and 76, respectively. However, these 2 patients had prior transfusions, and their high cPRA levels impeded the ability to find a suitable donor. In addition, one of these 2 patients had advanced chronic renal insufficiency and was seeking listing for combined lung and kidney transplants. These 2 patients were active on the lung transplant wait list for a few weeks before succumbing to severe acute exacerbations of their disease.

**Fig 2:**
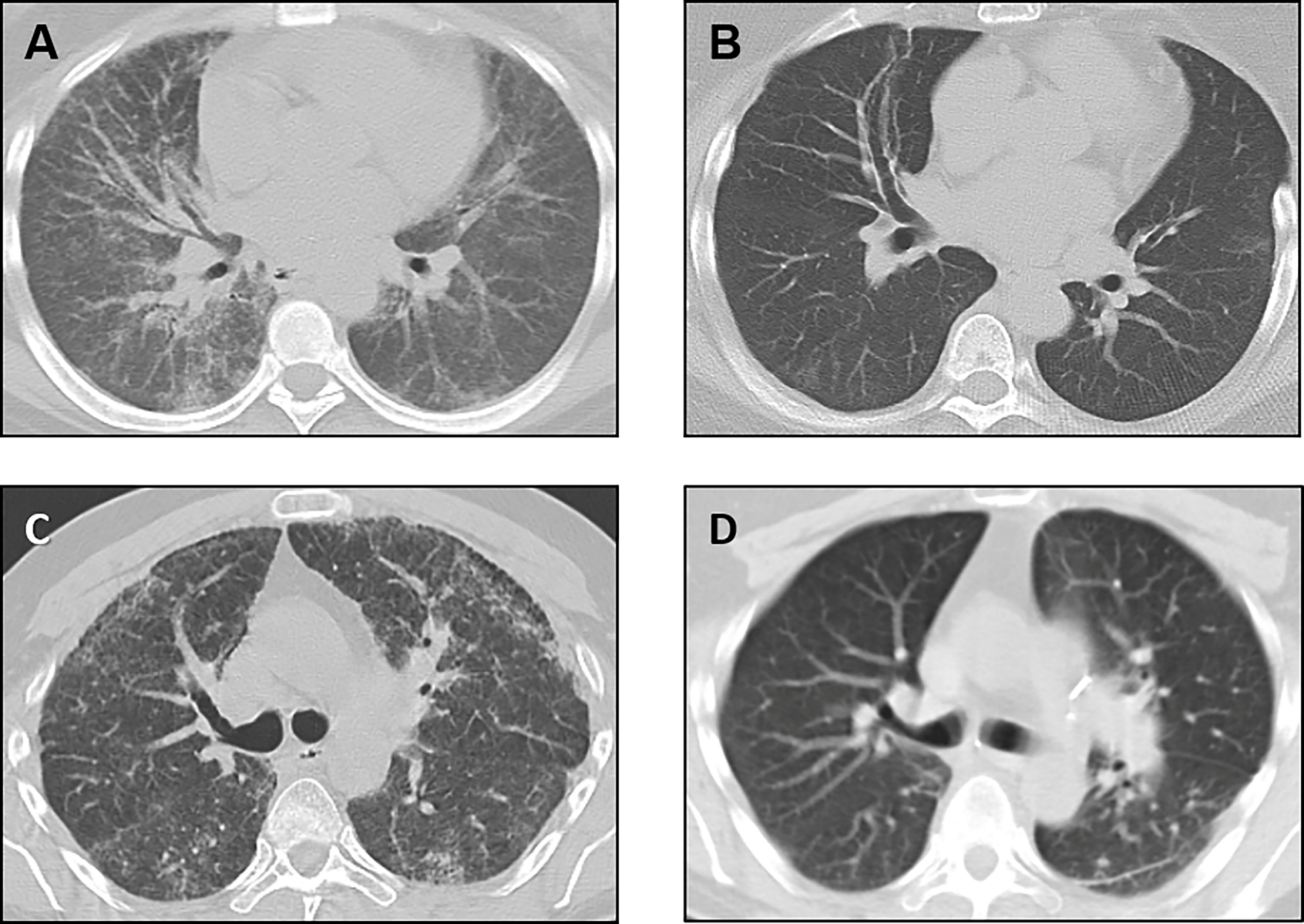
Chest computed tomography scans in Hermansky-Pudlak syndrome pulmonary fibrosis patients before and after lung transplantation. Representative computed tomography scan images of the chest from one patient with Hermansky-Pudlak syndrome pulmonary fibrosis showing diffuse bilateral interstitial infiltrates at the time of referral for lung transplantation (A) and 6 years after bilateral lung transplant surgery (B). Representative computed tomography scan images of the chest from another patient with Hermansky-Pudlak syndrome pulmonary fibrosis showing bilateral pulmonary fibrosis before lung transplantation (C) and 1.5 years after bilateral lung transplant surgery (D). Post-operative surgical changes are found, but there is no radiographic evidence of recurrence of Hermansky-Pudlak syndrome pulmonary fibrosis in the lung allografts.

Three of 6 patients successfully underwent bilateral lung transplantation at the same center. One patient was initially evaluated 9 months before receiving a bilateral lung transplant at this regional lung transplantation center. Pre-transplant evaluation included consultations with pulmonary medicine, hematology, gastroenterology, and dermatology. Forced vital capacity was 35% of predicted, and the patient was receiving continuous supplemental oxygen. The PAP was 27 mm Hg; the LAS was 41. The patient did not have a history of excessive bleeding or unusual bruising, and had never received blood product transfusions. There was no history of malignancy or colitis, and gastroenterology was consulted only for routine colon cancer screening. The patient received desmopressin by continuous intravenous infusion for approximately 14 hours in the peri-operative period as prophylaxis against bleeding. Bilateral lung transplantation was performed without immediate surgical complications or excessive intra-operative bleeding, and the patient did not receive a platelet transfusion during or after surgery. Routine surveillance bronchoscopies with transbronchial biopsies were performed using infusion of desmopressin as prophylaxis against bleeding. This patient was doing well more than 6 years after transplant, and high-resolution computed tomography scan showed no recurrence of HPS pulmonary fibrosis (Fig 2A and Fig 2B). Lung function was stable with an FVC of 77% of predicted, and there was no evidence of chronic lung allograft dysfunction.

A second patient underwent bilateral lung transplant surgery. The PAP was 37 mm Hg; the LAS was 65. Given this patient’s lack of prior episodes of excessive bleeding and *in vitro* platelet aggregation results, prophylactic intravenous desmopressin was not administered prior to surgery. However, platelets were made available for possible surgical bleeding. The patient experienced mild bleeding during transplant surgery, which was treated successfully with transfusion of several units of leuko-reduced platelets. This patient developed one episode of acute rejection (A1B0) with declining pulmonary function test measurements 1 month after transplantation. Lung function improved following a course of pulsed intravenous steroids. This patient was clinically stable with an FVC of 57% predicted approximately 1.5 years after lung transplantation. Routine clinical evaluations post-transplantation did not demonstrate evidence of chronic lung allograft dysfunction or recurrence of HPS lung disease (Fig 2C and Fig 2D). Surveillance bronchoscopies with transbronchial biopsies were performed, and effective hemostasis was achieved without desmopressin.

A third patient was referred to a regional lung transplantation center with an FVC of 46% of predicted. The patient did not have a history of excessive bleeding or prior blood product transfusions. There was no detectable reactivity to Class I or Class II antibodies, and cPRA was zero. Routine screening revealed no occult malignancy or other contraindications for transplantation. The patient experienced an unexpected, rapid decline in pulmonary status, received veno-venous extracorporeal membrane oxygenation for 11 days, and was anti-coagulated with a therapeutic dose of intravenous heparin infusion. The PAP was 74 mm Hg; the LAS was 90. Bilateral lung transplantation was performed, and mild diffuse surgical bleeding was successfully treated with prothrombin complex concentrate, platelet transfusion, and intravenous desmopressin. The post-operative course was notable for deep venous thromboses at central venous catheter sites; these were treated with enoxaparin, and subsequent development of retroperitoneal hemorrhage that was treated with aminocaproic acid and temporary discontinuation of anti-coagulation. Enoxaparin at half of standard therapeutic anti-coagulation dosage was re-started after the retroperitoneal hemorrhage stabilized. Although deep venous thromboses remained after 3 months of therapy with lower dose enoxaparin, the patient did not experience clinical evidence of thrombus extension or emboli. Notably, this patient did not develop acute rejection, and FVC was 46% predicted and slowly improving with good quality of life 4 months after lung transplantation. A surveillance bronchoscopy with transbronchial biopsies was performed after transfusion of 1 unit of platelets, and another was successfully performed without desmopressin.

### Query of OPTN database

A query of the OPTN database revealed that 16 patients with HPS pulmonary fibrosis were listed for lung transplant from January 2005 through August 2014. Seven patients were removed from the waitlist without receiving a lung transplant. Ten lung transplants were performed in 9 patients with HPS pulmonary fibrosis, including 2 of 3 recipients of bilateral lung transplants in this report. Wait times for patients with HPS pulmonary fibrosis on the lung transplant list ranged from 13 to 507 days, with a median of 84.5 days.

## Discussion

Hermansky-Pudlak syndrome is an autosomal recessive disease caused by mutations in one of 10 reported *HPS* genes [4–12]. Clinical manifestations of HPS include oculocutaneous albinism, a bleeding diathesis, and colitis [2, 3]. Pulmonary fibrosis develops in adults with HPS-1, HPS-2, and HPS-4 as well as in children with HPS-2 [2, 13–15]. Pulmonary fibrosis is a major cause of mortality in adults with HPS-1. Although therapeutic trials have been conducted for HPS pulmonary fibrosis, no drugs are approved to treat this fatal disease [16, 17]. However, lung transplantation has been performed in patients with advanced HPS pulmonary fibrosis, and this intervention remains the only viable option to prolong life expectancy in individuals with HPS pulmonary fibrosis. According to a database administered by UNOS, 9 patients with HPS pulmonary fibrosis received lung transplants in the United States from 2005 to 2014. These patients include 2 of our 3 patients with HPS pulmonary fibrosis, who are clinically well and do not have radiographic evidence of recurrent disease 1.5 and 6 years after receiving bilateral lung transplantations.

Patients with HPS have a platelet storage pool deficiency, which causes bleeding due to functional platelet abnormalities. Analysis of platelets by whole mount electron microscopy demonstrates absent delta granules, and our 6 patients did not have detectable delta granules in their platelets. Routine hematology test results are often normal, including platelet counts, prothrombin time and activated partial thromboplastin time. Bleeding time can be prolonged, and *in vitro* platelet analysis can demonstrate an abnormal secondary aggregation response in HPS [23].

The bleeding diathesis of HPS is commonly treated with desmopressin or platelet transfusions. The mechanism of action of desmopressin on platelets has not been fully elucidated, and the clinical response to desmopressin in patients with HPS can be variable [24–26]. However, desmopressin has been reported to ameliorate bleeding in some patients with HPS. The lung transplant recipients in this report did not have a history of easy bruising or unusual bleeding. One patient received intravenous desmopressin prior to bilateral lung transplantation and was not transfused with platelets. Another patient, who did not receive desmopressin, received platelets for mild bleeding during transplant surgery, and a third patient, who was anti-coagulated for extracorporeal membrane oxygenation therapy, received intraoperative prothrombin complex concentrate, desmopressin and platelets. In prior reports of patients with HPS pulmonary fibrosis undergoing lung transplantation, one patient received platelets before surgery, and another patient did not receive desmopressin or platelets before or during surgery [18, 20]. Effective hemostasis was achieved in all of these cases.

These clinical outcomes with disparate management for bleeding indicate that developing individualized therapeutic plans for management of potential peri-operative bleeding is appropriate for patients with HPS pulmonary fibrosis. Desmopressin infusion or platelet transfusion could be considered for prophylaxis against surgical bleeding. Alternatively, observation for bleeding without administering prophylactic treatment may be an option in select patients. We recommend that platelets be immediately available for all patients with HPS in anticipation of possible peri-operative bleeding, even for those who receive desmopressin for prophylaxis.

The three lung transplant recipients in this report did not have a history of transfusions. However, two other patients with HPS pulmonary fibrosis who previously received blood products developed alloimmunization and did not receive lung transplants. Their cPRA scores were high, and one patient’s extensive degree of antibody reactivity substantively limited potential donors. These cases highlight the need to limit alloimmunization and demonstrate the importance of performing genetic testing to identify patients with HPS at high risk for pulmonary fibrosis. Clinical approaches to avoid alloimmunization include minimizing blood product transfusions through the use of procoagulant drugs, transfusing leukoreduced single donor platelets or blood products, and, in certain situations, transfusing HLA-matched platelets [27, 28]. Given the potential need for blood product transfusions throughout their lives, patients with HPS at risk for pulmonary fibrosis should be evaluated by hematologists to develop individual plans for management of bleeding issues that limit alloimmunization and optimize their candidacy for potential future lung transplants. In addition, perioperative desensitization has been safely performed in sensitized lung transplant candidates, and may be a possible therapeutic strategy for sensitized patients with severe HPS pulmonary fibrosis [29].

Although HPS patients tend to bleed, those with progressive pulmonary fibrosis should be evaluated for lung transplantation, and extracorporeal membrane oxygenation can be used as a temporizing therapeutic intervention. Given the functional platelet defect related to HPS, consultation with a hematologist is recommended to address hematologic issues that may be associated with utilizing extracorporeal membrane oxygenation in patients with HPS. While the surgical outcome and survival of these 3 patients with HPS pulmonary fibrosis are similar to those in patients who undergo bilateral lung transplantation for idiopathic pulmonary fibrosis, further studies are needed to assess the clinical outcome of patients with HPS pulmonary fibrosis [30]. The ages of these patients and their lack of medical co-morbidities may have contributed to their clinical outcome. Thus, although donor lungs are scarce, patients with HPS pulmonary fibrosis are suitable candidates for bilateral lung transplants.

### Conclusions

Lung transplantation has been successfully performed in patients with HPS pulmonary fibrosis resulting in good clinical outcomes and long-term survival post-transplant in these 3 recipients without a history of excessive bleeding. However, the outcome of patients with HPS and a history of severe bleeding is unknown. Despite a tendency to bleed due to a platelet storage pool defect, effective hemostasis can be achieved in patients with HPS, and extracorporeal membrane oxygenation with anti-coagulation could be considered as temporary therapy for suitable patients with end-stage HPS pulmonary fibrosis. HPS is a rare disorder, and the small number of patients in this report is a limitation. Although the results should be interpreted with caution, these cases of HPS pulmonary fibrosis indicate that early referral to lung transplantation centers is prudent and highlight the presence of anti-HLA antibodies as a possible barrier to lung transplantation. Thus, implementing strategies to limit alloimmunization is important to optimize these patients’ future candidacy for lung transplantation.

## Supporting information

S1 Table Initial patient lung function and blood count tests. (DOCX)

## Abbreviations

cPRA: Calculated Panel of Reactive Antibodies
FVC: forced vital capacity
HPS: Hermansky-Pudlak syndrome
LAS: lung allocation score
NIH: National Institutes of Health
OPTN: Organ Procurement and Transplantation Network
PAP: pulmonary artery systolic pressure
