# Supplementary material for: Clinical management and outcomes of patients with Hermansky-Pudlak syndrome pulmonary fibrosis evaluated for lung transplantation
Source: PLoS One. 2018 Mar 16;13(3):e0194193. doi: 10.1371/journal.pone.0194193 (PMC5856338; doi:10.1371/journal.pone.0194193)
Supplement: S1 Table — (DOCX) [file pone.0194193.s001.docx]

**Supplemental Table 1: Initial patient lung function and blood count tests**

|  | Patient 1 | Patient 2 | Patient 3 | Patient 4 | Patient 5 | Patient 6 |
| --- | --- | --- | --- | --- | --- | --- |
| FVC (% predicted) | 59 | 46 | 58 | 35 | 46 | 14 |
| FEV_1_ (% predicted) | 64 | 53 | 66 | 39 | 50 | 16 |
| TLC (% predicted) | 53 | 53 | 72 | 36 | 47 | 23 |
| DLCO (% predicted) | 34 | 35 | 35 | 40 | 58 | unable |
| 6MWT (m) | 592 | 375 | 427 | 254 | 558 | 134 |
| HR post (per min) | 119 | 132 | 138 | 121 | 138 | 128 |
| O_2_ sat post (%) | 87 | 83 | 96 | 82* | 93 | 93* |
| WBC (K/uL) | 7.1 | 6.9 | 7.0 | 7.4 | 6.3 | 7.9 |
| PMN (K/uL) | 5.0 | 5.2 | 4.4 | 5.0 | 3.6 | 4.3 |
| Lymphocyte (K/uL) | 1.5 | 1.1 | 1.8 | 1.2 | 2.0 | 2.0 |
| Monocyte (K/uL) | 0.47 | 0.45 | 0.62 | 0.96 | 0.40 | 0.69 |
| Eosinophil (K/uL) | 0.20 | 0.16 | 0.26 | 0.19 | 0.21 | 0.90 |
| HGB (g/dL) | 15.6 | 13.3 | 13.2 | 9.1 | 12.3 | 13.0 |
| HCT (%) | 46.8 | 40.7 | 40.6 | 29.3 | 37.8 | 42.4 |
| MCV (fL) | 87.6 | 89.6 | 82.5 | 74.2 | 79.2 | 88.6 |
| RDW (%) | 33.3 | 13.3 | 14.7 | 17.5 | 13.4 | 13.6 |

FVC, forced vital capacity

FEV_1_, forced expiratory volume in 1 second

TLC, total lung capacity

DLCO, diffusion capacity

6MWT, 6-minute walk test distance

HR post, heart rate after 6-minute walk test

O_2_ sat post, oxygen saturation after 6-minute walk test

WBC, white blood cell

PMN, polymorphonuclear leukocyte

HGB, hemoglobin

HCT, hematocrit

MCV, mean corpuscular volume

RDW, red blood cell distribution width

* supplemental oxygen, 3 liters/minute
